# Supplementary material for: Transcriptional Analysis of Effusion-Based Lymphoma Supports a Post-Germinal Center Origin and Specific Inflammatory Signal Background
Source: Cancers (Basel). 2025 Sep 12;17(18):2978. doi: 10.3390/cancers17182978 (PMC12468710; doi:10.3390/cancers17182978)
Supplement: Supplementary file 1 [file cancers-17-02978-s001.zip › cancers-3857702-supplementary.pdf]

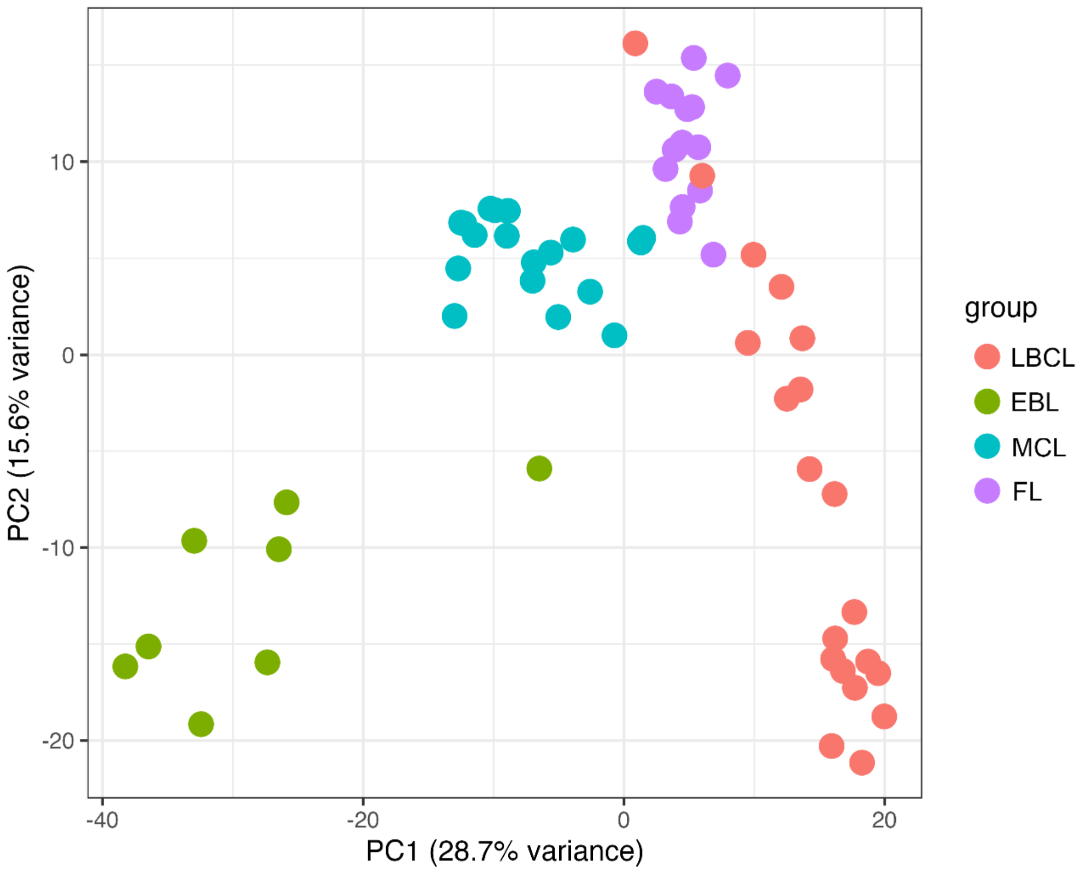

Supplementary Figure S1. Principal component analysis (PCA) was performed on log-CPM values from the gene-expression analysis using the PanCancer Immune Profiling Panel (NanoString Technologies). Each point represents one sample, colored by group. Percentages on the axes represent variance captured by the first two principal components, based on the expression of genes included in the NanoString PanCancer panel. LBCL: Large B-cell lymphoma, EBL: Effusion-based lymphoma, MCL: Mantle cell lymphoma, and FL: Follicular lymphoma.

Case	Age	Sex	Effusion Site(s)	Fluid Overload Risk	HIV	HCV	Treatment	Response	Follow-up (months)	Outcome
1	61	M	Pleural	CAD, ESRD	1	0	R-CHOP ×4 +R-CEOP ×3	Complete	39	Deceased
2	65	M	Pleural	NA	NA	NA	NA	NA	NA	NA
3	72	M	Pericardial	CHF, CKD	0	0	None	Complete	15.6	Deceased
4	84	F	Pericardial	None	0	0	Rituximab	Complete	86.9	Alive
5	91	M	Pleural, Pericardial	CHF	0	0	Drainage only	Persistent	5.3	Deceased
6	88	M	Pleural	CHF	0	0	None	Persistent	0.2	Deceased
7	90	M	Pleural	CHF	0	0	Drainage +Rituximab ×1	Persistent	3.2	Deceased
8	90	F	Pleural, Pericardial	CHF, HTN, HT	NA	NA	None	Persistent	2	Deceased

Supplementary Table S1. Detailed clinical characteristics of the included EBL cases. Abbreviations: coronary artery disease (CAD), end-stage renal disease (ESRD), chronic heart failure (CHF), chronic kidney disease (CKD), hypertension (HTN), hypothyroidism (HT).

Pathway	score	Overlapping genes
Plasma cell differentiation	0.50	HLA-A, HLA-B, HLA-C, HLA-E, HLA-G, RPS6, TXNIP, UBC
HLA presentation	0.42	HLA-A, HLA-B, HLA-C, HLA-E, HLA-G
High-grade signature	0.36	CD40, CD44, CTSH, NFKBIA, STAT3
Quiescence/low proliferation	0.21	CD44, HLA-E, HLA-G, ICAM3, IFNAR2, IKBKB, IL10RA, PECAM1, PTPRC, SELL, SELPLG, STAT6, TNFSF10, TXNIP, UBC
ABC_gt_GCB_PMBL_MCL_BL_U133AB (ABC-type)	0.19	BATF, BTLA, CD44, ENTPD1, HCK, IL10, IRF4, POU2F2, STAT3, TFRC
IRF4/MUM1 signaling	0.18	BCL2, CASP3, CCL3, IRF4, SLAMF7, TNFRSF12A, VEGFA
CD8 T-cell effector	0.17	CCL4, GZMH, HLA-DPA1, IFI16, IL7R, KLRD1, NFKBIA
ABC_gt_GCB_Affy	0.20	BATF, BCL2, ENTPD1, IL16, IRF4

Supplementary Table S2. Enrichment of the genes identified using the reference SignatureDB for DLBCL.

Pathway ID	Pathway ID	overlap	Adjusted p Value	Overlapping genes
hsa04060	Cytokine-cytokine receptor interaction	66/225	1.71E-44	CCL1/CCL11/CCL13/CCL15/CCL16/CCL19/CCL20/CCL22/CCL24/CCL25/CCL26/CCL27/CCL28/CCL3/CCR3/CCR9/CD27/CD4/CD40LG/CSF2RB/CSF3/CXCL6/CXCL9/CXCR1/CXCR2/IFNA1/IFNA17/IFNA2/IFNA7/IFNA8/IFNAR1/IFNB1/IFNGR1/IFNL2/IL11/IL12B/IL13/IL13RA2/IL17A/IL17F/IL18RAP/IL19/IL1A/IL1RL1/IL1RL2/IL2/IL21/IL22/IL23A/IL23R/IL24/IL25/IL26/IL27/IL32/IL4/IL5RA/LIF/LTBR/TGFB2/TNFRSF14/TNFRSF9/TNFSF11/TNFSF12/TNFSF18/XCR1
hsa05321	Antigen/presentation T-cells	27/225	4.12E-25	GATA3/HLA-DMA/HLA-DMB/HLA-DOB/HLA-DRA/HLA-DRB3/IFNGR1/IL12B/IL13/IL17A/IL17F/IL18RAP/IL1A/IL2/IL21/IL22/IL23A/IL23R/IL4/NFATC1/RORC/STAT3/STAT6/TBX21/TGFB2/TLR2/TLR4
hsa04630	JAK-STAT signaling	36/225	5.59E-23	AKT3/BCL2/CSF2RB/CSF3/EP300/IFNA1/IFNA17/IFNA

				2/IFNA7/IFNA8/IFNAR1/IFNB1/IFNGR1/IFNL2/IL11/IL12B/IL13/IL13RA2/IL19/IL2/IL21/IL22/IL23A/IL23R/IL24/IL26/IL27/IL4/IL5RA/JAK2/LIF/PIK3CD/STAT2/STAT3/STAT5B/STAT6
hsa05169	NF-κB/B-cell activation signaling	38/225	4.35E-22	AKT3/BCL2/BTK/CD44/HLA-A/HLA-DMA/HLA-DMB/HLA-DOB/HLA-DRA/HLA-DRB3/HLA-G/ICAM1/IFNA1/IFNA17/IFNA2/IFNA7/IFNA8/IFNAR1/IFNB1/IKBKB/IKBKE/IRF3/IRF7/MAP3K7/MAPK14/MAVS/PIK3CD/PSMD7/RUNX3/STAT2/STAT3/SYK/TAB1/TAPBP/TLR2/TNFAIP3/TP53/TRAF2
hsa04640	Immune cell development	27/225	4.00E-20	CD1E/CD22/CD24/CD38/CD4/CD44/CD55/CD59/CD7/CD9/CSF3/HLA-DMA/HLA-DMB/HLA-DOB/HLA-DRA/HLA-DRB3/IL11/IL1A/IL4/IL5RA/ITGA2/ITGA2B/ITGA5/ITGAM/KIT/MS4A1/TFRC
hsa05152	Innate immune activation: macrophage/TLR response	34/225	6.17E-20	AKT3/BCL2/CASP10/CD74/CEBPB/CLEC7A/EP300/FCGR2A/FCGR2B/HLA-DMA/HLA-DMB/HLA-

				DOB/HLA-DRA/HLA-DRB3/IFNA1/IFNA17/IFNA2/IFNA7/IFNA8/IFNB1/IFNGR1/IL12B/IL1A/IL23A/ITGAM/ITGAX/ITGB2/JAK2/LBP/MAPK14/SYK/TGFB2/TLR2/TLR4
hsa04620	Toll-like receptor signaling	27/225	3.86E-19	AKT3/CCL3/CXCL9/IFNA1/IFNA17/IFNA2/IFNA7/IFNA8/IFNAR1/IFNB1/IKBKB/IKBKE/IL12B/IRF3/IRF7/LBP/LY96/MAP2K2/MAP3K7/MAPK14/PIK3CD/SPP1/STAT2/TAB1/TLR2/TLR3/TLR4
hsa05171	Interferon & hyperinflammatory immune response	36/225	4.82E-18	C1QA/C1R/C1S/C5/C6/C8A/C8B/C9/CSF3/FCGR2A/IFNA1/IFNA17/IFNA2/IFNA7/IFNA8/IFNAR1/IFNB1/IKBKB/IKBKE/IL12B/IL2/IRF3/MAP3K7/MAPK14/MASP1/MAVS/MBL2/NRP1/PIK3CD/RPS6/STAT2/STAT3/SYK/TLR2/TLR3/TLR4
hsa04659	Th17 polarization	26/225	4.82E-18	CD4/GATA3/HLA-DMA/HLA-DMB/HLA-DOB/HLA-DRA/HLA-DRB3/IFNGR1/IKBKB/IL17A/IL17F/IL2/IL21/IL22/IL23A/IL23R/IL27/IL4/JAK2/MAPK14/NFATC1/RORC/STAT3/STAT5B/STAT6/TBX21

hsa04610	Complement activation	22/225	8.14E-16	A2M/C1QA/C1R/C1S/C4BPA/C5/C6/C8A/C8B/C9/CD55/CD59/CFI/CLU/F12/ITGAM/ITGAX/ITGB2/MASP1/MBL2/PLAU/SERPINB2
hsa05140	Macrophage activation & inflammation	20/225	1.52E-14	FCGR2A/HLA-DMA/HLA-DMB/HLA-DOB/HLA-DRA/HLA-DRB3/IFNGR1/IL12B/IL1A/IL4/ITGAM/ITGB2/JAK2/MAP3K7/MAPK14/PTGS2/TAB1/TGFB2/TLR2/TLR4
hsa04658	Th1/Th2 polarization	20/225	3.90E-13	CD4/GATA3/HLA-DMA/HLA-DMB/HLA-DOB/HLA-DRA/HLA-DRB3/IFNGR1/IKBKB/IL12B/IL13/IL2/IL4/JAK2/MAPK14/NFATC1/RUNX3/STAT5B/STAT6/TBX21

Supplementary Table S3. Enrichment of the genes identified using the reference KEGG signaling pathways.
